# Supplementary material for: Bond performance between hybrid fiber-reinforced concrete and BFRP bars under freeze-thaw cycle
Source: PLoS One. 2024 May 13;19(5):e0303327. doi: 10.1371/journal.pone.0303327 (PMC11090331; doi:10.1371/journal.pone.0303327)
Supplement: Attachment — Submitted filename: Response to Reviewers.docx [file pone.0303327.s002.docx]

**Answer to Academic editor:**

**Journal requirements:**

1. When submitting your revision, we need you to address these additional requirements. Please ensure that your manuscript meets PLOS ONE's style requirements, including those for file naming. The PLOS ONE style templates can be found at https://journals.plos.org/plosone/s/file?id=wjVg/PLOSOne_formatting_sample_main_body.pdf and https://journals.plos.org/plosone/s/file? id=ba62/PLOSOne_formatting_sample_title_authors_affiliations.pdf.

R: Thanks for your suggestion. I have revised the manuscript to meet PLOS ONE’s style requirements.

2. We suggest you thoroughly copyedit your manuscript for language usage, spelling, and grammar. If you do not know anyone who can help you do this, you may wish to consider employing a professional scientific editing service.

Whilst you may use any professional scientific editing service of your choice, PLOS has partnered with both American Journal Experts (AJE) and Editage to provide discounted services to PLOS authors. Both organizations have experience helping authors meet PLOS guidelines and can provide language editing, translation, manuscript formatting, and figure formatting to ensure your manuscript meets our submission guidelines. To take advantage of our partnership with AJE, visit the AJE website (http://learn.aje.com/plos/) for a 15% discount off AJE services. To take advantage of our partnership with Editage, visit the Editage website (www.editage.com) and enter referral code PLOSEDIT for a 15% discount off Editage services. If the PLOS editorial team finds any language issues in text that either AJE or Editage has edited, the service provider will re-edit the text for free.

Upon resubmission, please provide the following: The name of the colleague or the details of the professional service that edited your manuscript A copy of your manuscript showing your changes by either highlighting them or using track changes (uploaded as a *supporting information* file) A clean copy of the edited manuscript (uploaded as the new *manuscript* file)”.

R: As the Reviewer's good instruction, I have tried our best to revise the English of the whole manuscript carefully. In order to make the whole manuscript better understanding, I have revised some long sentences into short sentences. Meanwhile, I also have asked some colleagues who are skilled of English language to help us for checking the English. I hope that the language is now acceptable for the next review process. Finally, if the revised article is still not up to the standard of your magazine, I would like to use the organisation you partner with to revise my article to meet your requirements Special thanks to you for your good comments !

3. Please note that PLOS ONE has specific guidelines on code sharing for submissions in which author-generated code underpins the findings in the manuscript. In these cases, all author-generated code must be made available without restrictions upon publication of the work. Please review our guidelines at https://journals.plos.org/plosone/s/materials-and-softwaresharing#loc-sharing-code and ensure that your code is shared in a way that follows best practice and facilitates reproducibility and reuse.

R: Thanks for your comment. The relevant data has been uploaded as Supporting Information files.

4. Thank you for stating the following financial disclosure: [This work was supported by a grant from the National Natural Science Foundation of China (No. 51938009).].  Please state what role the funders took in the study. If the funders had no role, please state: ""The funders had no role in study design, data collection and analysis, decision to publish, or preparation of the manuscript."" If this statement is not correct you must amend it as needed. Please include this amended Role of Funder statement in your cover letter; we will change the online submission form on your behalf.

R: Thanks for your suggestion. In the cover letter, I have explicitly stated that the funders had no role in study design, data collection and analysis, decision to publish, or preparation of the manuscript.

5. Thank you for stating the following in the Acknowledgments Section of your manuscript: [This work was supported by a grant from the National Natural Science Foundation of China (No. 51938009).] We note that you have provided funding information that is not currently declared in your Funding Statement. However, funding information should not appear in the Acknowledgments section or other areas of your manuscript. We will only publish funding information present in the Funding Statement section of the online submission form. Please remove any funding-related text from the manuscript and let us know how you would like to update your Funding Statement. Currently, your Funding Statement reads as follows: [This work was supported by a grant from the National Natural Science Foundation of China (No. 51938009).]. Please include your amended statements within your coverletter; we will change the online submission form on your behalf.

R: Thanks for your suggestion. I have already removed the information related to funding from the manuscript.

6. We note that your Data Availability Statement is currently as follows: [All relevant data are within the manuscript and its Supporting Information files.] Please confirm at this time whether or not your submission contains all raw data required to replicate the results of your study. Authors must share the “minimal data set” for their submission. PLOS defines the minimal data set to consist of the data required to replicate all study findings reported in the article, as well as related metadata and methods (https://journals.plos.org/plosone/s/data-availability#locminimal-data-set-definition).

For example, authors should submit the following data:

- The values behind the means, standard deviations and other measures reported;

- The values used to build graphs;

- The points extracted from images for analysis.

Authors do not need to submit their entire data set if only aportion of the data was used in the reported study.

If your submission does not contain these data, please either upload them as Supporting Information files or deposit them to a stable, public repository and provide us with the relevant URLs, DOIs, or accession numbers. For a list of recommended repositories, please see https://journals.plos.org/plosone/s/recommendedrepositories.

If there are ethical or legal restrictions on sharing a deidentified data set, please explain them in detail (e.g., data contain potentially sensitive information, data are owned by a third-party organization, etc.) and who has imposed them (e.g., an ethics committee). Please also provide contact information for a data access committee, ethics committee, or other institutional body to which data requests may be sent. If data are owned by a third party, please indicate howothers may request data access.

R: Thanks for your comment. The relevant data has been uploaded as Supporting Information files.

7. PLOS requires an ORCID iD for the corresponding author in Editorial Manager on papers submitted after December 6th, 2016. Please ensure that you have an ORCID iD and that it is validated in Editorial Manager. To do this, go to‘Update my Information’ (in the upper left-hand corner of the main menu), and click on the Fetch/Validate link next to the ORCID field. This will take you to the ORCID site and allow you to create a new iD or authenticate a pre-existing iD in Editorial Manager. Please see the following video for instructions on linking an ORCID iD to your Editorial Manager account: https://www.youtube.com/watch?v=_xcclfuvtxQ".

R: Thanks for your comment. I have updated and validated my ORCID in Editorial Manager.

8. We note that Figure(s) 2, 3a, 3b, 5, 6, 7, 8a, 8b, 9a and 9b in your submission contain copyrighted images. All PLOS content is published under the Creative Commons Attribution License (CC BY 4.0), which means that the manuscript, images, and Supporting Information files will be freely available online, and any third party is permitted to access, download, copy, distribute, and use these materials in anyway, even commercially, with proper attribution. For more information, see our copyright guidelines: http://journals.plos.org/plosone/s/licenses-and-copyright.

We require you to either (1) present written permission from the copyright holder to publish these figures specifically under the CC BY 4.0 license, or (2) remove the figures from your submission:
 a. You may seek permission from the original copyright holder of Figure(s) 2, 3a, 3b, 5, 6, 7, 8a, 8b, 9a and 9b to publish the content specifically under the CC BY 4.0 license.

We recommend that you contact the original copyright holder with the Content Permission Form (http://journals.plos.org/plosone/s/file?id=7c09/contentpermission-form.pdf) and the following text:“I request permission for the open-access journal PLOS ONE to publish XXX under the Creative Commons Attribution License (CCAL) CC BY 4.0 (http://creativecommons.org/licenses/by/4.0/).

Please be aware that this license allows unrestricted use and distribution, even commercially, by third parties. Please reply and provide explicit written permission to publish XXX under a CC BY license and complete the attached form.” Please upload the completed Content Permission Form or other proof of granted permissions as an ""Other"" file with your submission.

In the figure caption of the copyrighted figure, please include the following text: “Reprinted from [ref] under a CC BY license, with permission from [name of publisher], original copyright [original copyright year].”

b. If you are unable to obtain permission from the original copyright holder to publish these figures under the CC BY 4.0 license or if the copyright holder’s requirements are incompatible with the CC BY 4.0 license, please either i) remove the figure or ii) supply a replacement figure that complies with the CC BY 4.0 license. Please check copyright information on all replacement figures and update the figure caption with source information. If applicable, please specify in the figure caption text when a figure is similar but not identical to the original image and is therefore for illustrative purposes only.

R: Thank you for your advice. Figure (s) 2, 3a, 3b, 5, 6, 7, 8a, 8b, 9a and 9b all belong to the real photos I took during the experiment, and do not have copyright issues with other organizations or individuals. I still provide a Content Permission Form about myself.

**Answer to Reviewer #1:**

1. There are several references speaking about and interrupting with models that are not mentioned here El-Nemr, A., Ahmed, E. A., Barris, C., Joyklad, P., Hussain, Q., & Benmokrane, B. (2023). Bond performance of fiber reinforced polymer bars in normal- and high-strength concrete. In Construction and Building Materials (Vol. 393, p. 131957). Elsevier BV. https://doi.org/10.1016/j.conbuildmat.2023.131957 F. Faqih, T. Zayed, E. Soliman, Factors and Defects Analysis of Physical and Environmental Condition of Buildings, J. Build. Pathol. Rehabilit. 5 (2020) 1–15.

R: Grateful for your valuable feedback, I acknowledge the meticulous scientific detail and substantial research significance embedded within the two referenced articles. My validation process involved scrutinizing the models delineated in these papers, leveraging data gleaned from my experimental study. Regrettably, the model’s applicability to the bond performance between BFRP reinforcement bars and hybrid fiber-reinforced concrete post freeze-thaw cycles proved inadequate. Nonetheless, I dutifully cite these seminal works to disseminate their pivotal findings.

[R1] El-Nemr A, Ahmed EA, Barris C, Joyklad P, Hussain Q, Benmokrane B. Bond performance of fiber reinforced polymer bars in normal- and high-strength concrete. Constr Build Mater. 2023;393:131957. doi: 10.1016/j.conbuildmat.2023.131957.

[R2] Faqih F, Zayed T, Soliman E. Factors and defects analysis of physical and environmental condition of buildings. J. Build. Pathol. Rehabilit. 2020;5(1):19. doi: 10.1007/s41024-020-00084-0.

(See Page 28, Lines 573-575; Pages 28, Lines 567-569)

2. The figures are not in its position and need to be clearer.

R: Appreciation is extended to the reviewer for their valuable suggestion. As per the submission guidelines outlined by the PLOS ONE journal, figures are expressly excluded from the main manuscript file. Instead, each figure must be meticulously prepared and submitted as an individual file. Notably, the image clarity has been further enhanced to align with the stringent requirements stipulated by the PLOS ONE journal.

3. More analysis is required.

R: I sincerely thank the reviewer for careful reading. I have incorporated various analyses, including the impact analysis of hybrid fibers on bond strength. As follows: “Fig 11(b) shows a histogram of the effect of the incorporation of the hybrid fiber on bond strength. The bond strengths of hybrid fiber-reinforced concrete specimens were always higher than that of ordinary concrete specimens for the same number of freeze-thaw cycles. The bond strength of specimen C0.15B0.2 increased by 7.88% compared to that of specimen C0B0 under 0 freeze-thaw cycles. The bond strength of each specimen gradually decreased with an increasing number of freeze-thaw cycles. However, after 25 freeze-thaw cycles, the bond strength of specimen C0.15B0.2 remained 4.23% higher than that of specimen C0B0. Notably, the enhancement effect of hybrid fibers on bond strength diminished as the freeze-thaw cycle count increased. The effect of reinforcing toughness of the “fiber network” formed by the random distribution of CF and BF in concrete, which acted as a “micro-reinforcement”. The initial defects of concrete could be compensated by the BF bridged at cracks within the concrete. The elastic modulus and tensile properties of BF surpassed those of cellulose fibers. Consequently, BF could more effectively enhance the tensile strength of concrete and restrain the propagation of internal cracks. Simultaneously, CF possessed a unique porous structure and natural hydrophilicity, allowing it to absorb non-frozen pore solution and free water within the concrete, thereby reducing internal hydrostatic pressure [R3]. The uniform dispersion of CF and BF within the concrete matrix formed a three-dimensional spatial system. This not only elevated the overall tensile strength of concrete but also reduced the connectivity of internal pores, segmenting large pores into smaller ones and minimizing water infiltration. Consequently, concrete’s frost resistance was effectively improved, while bond performance was maximally preserved. However, as the number of freeze-thaw cycles increased, internal freeze-thaw damage and the count of interconnected cracks escalated, leading to greater water ingress into the concrete. The proportion of moisture absorbed by CF and the voids segmented by hybrid fibers diminishes. Additionally, the Ca(OH)_2_ produced during cement hydration dissolved in water. Water containing Ca(OH)_2_ was absorbed into the CF’s cavities, resulting in mineralization and a decline in the mechanical and durability properties of the fibers [R4]. With more freeze-thaw cycles, an increasing amount of Ca(OH)_2_ infiltrated the cavities, exacerbating fiber degradation. Consequently, the reinforcing effect of hybrid fibers gradually diminished. This phenomenon explained why, with an increasing number of freeze-thaw cycles, the reduction in bond strength between hybrid fiber-reinforced concrete and BFRP bars exceeded that observed in ordinary concrete-BFRP bond strength. New analysis on the bond-slip curve, which was “The bond stiffness of pull-out specimens slightly exceeded that of specimens subjected to 50 freeze-thaw cycles when the number of freeze-thaw cycles reached 75. This phenomenon could be attributed to the inclusion of certain inorganic solutes during the freeze-thaw test, which infiltrate the concrete [R5]. As the freeze-thaw cycle count increased, unhydrated cement particles within the concrete react with these solutes and water, resulting in secondary hydration reactions and the generation of additional hydration products. Consequently, the internal structure of the concrete became denser, leading to a marginal increase in elastic modulus. Although this effect is subtle, the slight rebound in concrete’s elastic modulus contributes to the slightly greater bond stiffness observed in pull-out specimens after 75 freeze-thaw cycles compared to those after 50 cycles.” In the section on bond-slip constitutive models, the importance of comparing model fitting results, the reason for the improvement of model fitting results with an increasing number of freeze-thaw cycles, and the conclusion that predicting the bond-slip curve of pull-out specimens after freeze-thaw cycles using the bond-slip constitutive model method will be more accurate have been added to the Revised Manuscript with Track Changes file.

[R3] Liu J, Lv C. Research Progress on Durability of Cellulose Fiber-Reinforced Cement-Based Composites. Int J Polym Sci. 2021;2021:1014531. doi: 10.1155/2021/1014531.

[R4] Ardanuy M, Claramunt J, Toledo Filho RD. Cellulosic fiber reinforced cement-based composites: A review of recent research. Constr Build Mater. 2015;79:115-128. doi: 10.1016/j.conbuildmat.2015.01.035.

[R5]	An M, Wang Y, Yu Z. Damage mechanisms of ultra-high-performance concrete under freeze–thaw cycling in salt solution considering the effect of rehydration. Constr Build Mater. 2019;198:546-552. doi: 10.1016/j.conbuildmat.2018.11.175.

(See Pages 32, Lines 638-639; Page 34, Lines 678-680; Pages 33, Lines 672-674;)

**Answer to Reviewer #2:**

1. In Section 2.1, regarding the description of the experimental materials, please provide additional details about the ribs of the BFRP, such as the rib spacing and rib height.

R: Thanks for your comment. In this experiment, I utilized BFRP bars with a rib spacing of 10 mm and a rib height of 1.4 mm. I have incorporated this information into Table 1, which outlines the material properties of BFRP bars in our revised manuscript. By doing so, I aim to enhance the clarity and precision of my research findings.”

(See Page 7, Lines 142)

2. In Section 3.1, if the author intends to illustrate the impact of freeze-thaw cycles on the failure mode of specimens, please supplement corresponding failure diagrams for comparison.

R: Thanks for your comment. As per the submission guidelines outlined by PLOS ONE, it is essential to refrain from including figures within the main manuscript file. Instead, each figure should be meticulously prepared and submitted as an individual file. Consequently, direct incorporation of images into the manuscript is not feasible. However, I have diligently uploaded two distinct files—one depicting the pull-out failure and the other illustrating the splitting failure. These images serve the purpose of providing a lucid representation of the experimental phenomena and significantly contribute to the analysis of failure modes. Their inclusion is specifically intended for comparative purposes.

3. In Section 3.2, based on the author's description, an increase in the number of freeze-thaw cycles is expected to result in a decrease in the bond-slip curve slope, i.e., the bond stiffness. Please explain the reason for the observed phenomenon where the bond stiffness of specimens subjected to 75 freeze-thaw cycles is greater than that of specimens subjected to 50 freeze-thaw cycles, both with and without fiber incorporation.

R: Thanks for your detailed analysis. Numerous studies have consistently demonstrated that freeze-thaw cycles can induce concrete spalling, increase porosity, and reduce the elastic modulus, ultimately leading to decreased bond stiffness. My research aligns with this overarching trend. Interestingly, irrespective of the inclusion of hybrid fibers, the pull-out specimens subjected to 75 freeze-thaw cycles exhibit slightly greater bond stiffness than those subjected to 50 cycles. The rationale behind this phenomenon lies in the freeze-thaw testing process, which introduces water-soluble inorganic substances into the concrete matrix. As the number of freeze-thaw cycles increases, unhydrated cement particles within the concrete undergo secondary hydration reactions with these water and inorganic components, resulting in the generation of additional hydration products [R5]. Consequently, the internal structure of the concrete becomes denser, leading to a marginal increase in the elastic modulus. However, it is crucial to note that this effect remains subtle. The slight rebound in the concrete’s elastic modulus contributes to the slightly higher bond stiffness observed in the pull-out specimens subjected to 75 cycles compared to those subjected to 50 cycles. I have diligently incorporated this description into the Revised Manuscript with Track Changes.

(See Page 14, Lines 291-301)

4. In Section 3.3, the correspondence between the two figures in Fig.11 and the analysis of the impact of freezethaw cycles and hybrid fibers on the bond strength between BFRP bars and concrete described in the article needs to be debated. Whether (a) it is more suitable to describe the effect of freeze-thaw cycles, and (b) it is more suitable to describe the effect of hybrid fiber addition.

R: I express my gratitude for your thoughtful consideration. After meticulous analysis, I recognize the significance of the correspondence between the two figures and their impact on bond strength. Upon reflection, I propose a strategic adjustment: swapping the order of the two figures. This modification enhances the conveyance of various influencing factors on bond strength. Consequently, in the recently uploaded image files, I have meticulously reversed the sequence of the two figures. The outcome is a more coherent and logically structured presentation within the article.

(See Page 24, Lines 530)

5. The significance of hybrid fibers requires clarification, and comparing only to specimens without fibers lacks persuasiveness. If possible, a comparison with specimens containing only BF and CF would enhance the completeness of the experimental results.

R: This study is an extension of the previous study. Zhu [R6] has previously discussed the advantages of the bonding performance between hybrid fiber recycled concrete and BFRP bars compared with the bonding performance between BF and CF single-doped concrete and BFRP bars after freeze-thaw cycles. Therefore, this test will no longer continue to explore the advantages of hybrid fibers and single-doped fibers in the bonding performance between ordinary concrete and BFRP bars. The purpose of this study was to continue to explore the influence of the incorporation of hybrid fibers on the bonding performance between ordinary concrete and BFRP bars after freeze-thaw cycles, and to improve the integrity of the research.

[R6] Zhu BH. Experimental Study on the Bond Behavior between BFRP Bars and Recycled Aggregate Concrete. Master Thesis, Liaoning University Of Technology, China, 2021. doi: 10.27211/d.cnki.glngc.2021.000002.

(See Page 32, Lines 647-649)

6. The significance of comparing the fitting results of the bond slip model needs further explanation.

R: Comparing the results of various bond-slip constitutive models provides insights into their applicability in our experimental context, ultimately allowing us to identify the most suitable bond-slip model. This determination holds significant engineering and scientific implications for predicting bond-slip curves. After contrasting the predictive outcomes of different models, we found that the Malvar model best represents the ascending segment of the bond-slip curve, while the continuous curve model is optimal for the descending segment. Notably, in the ascending segment, both the MBPE model and the Continuous Curve model exhibit improved fitting as the number of freeze-thaw cycles increases. Similarly, in the descending segment, there is an overall increasing trend in the fitting effect of each model for hybrid fiber-reinforced concrete as freeze-thaw cycles accumulate. This behavior can be attributed to the observed decrease in the slope of the bond-slip curve for both segments due to the reduction in concrete’s elastic modulus caused by freeze-thaw cycles. Notably, bond-slip constitutive models tend to exhibit better fitting performance for curves with lower slopes. Our findings confirm the high applicability of using bond-slip constitutive models to predict the bond-slip curves obtained in our experiment. Consequently, relative to bond-slip curves from pull-out specimens unaffected by freeze-thaw cycles, employing the bond-slip constitutive model for predicting bond-slip curves from pull-out specimens subjected to freeze-thaw cycles will yield more accurate results. We have incorporated these conclusions regarding the importance of model fitting comparisons, the enhancement of model fitting with increasing freeze-thaw cycles, and the accuracy of predicting bond-slip curves for freeze-thawed pull-out specimens using bond-slip constitutive models into the Revised Manuscript with Track Changes document.

7. In Section 3.5.2, including the replacement rate of recycled aggregate as one of the influencing factors, seems unnecessary. Please clarify the relevance of choosing this parameter and its significance, as it appears less related to the content of this paper.

R: I appreciate your thorough review. The decision to consider the replacement percentage of recycled aggregate as an influential factor stems from my study’s direct linkage to Zhu’s prior research [R6]. Given the substantial relevance between my current experiment and Zhu’s work, it becomes imperative to integrate his pull-out test data into my comprehensive database. In Zhu’s study, the issue of recycled aggregate replacement percentage was meticulously addressed. Consequently, I have diligently collected pull-out test data that includes relevant information on recycled aggregate replacement percentage. This strategic inclusion aims to significantly enhance the precision of my BP neural network predictions, thereby ensuring a scientifically robust and reliable forecasting approach.
